# Controlled-Deactivation CB1 Receptor Ligands as a Novel Strategy to Lower Intraocular Pressure

**DOI:** 10.3390/ph11020050

**Published:** 2018-05-22

**Authors:** Sally Miller, Shashank Kulkarni, Alex Ciesielski, Spyros P. Nikas, Ken Mackie, Alexandros Makriyannis, Alex Straiker

**Affiliations:** 1 The Gill Center for Biomolecular Science, The Department of Psychological and Brain Sciences, Indiana University, Bloomington, IN 47405, USA; sallmill@indiana.edu (S.M.); agciesie@umail.iu.edu (A.C.); kmackie@indiana.edu (K.M.); 2 Center for Drug Discovery, Departments of Chemistry & Chemical Biology and Pharmaceutical Sciences, Northeastern University, Boston, MA 02115, USA; kulkarni.sha@northeastern.edu (S.K.); s.nikas@northeastern.edu (S.P.N.); a.makriyannis@northeastern.edu (A.M.)

**Keywords:** cannabinoid, glaucoma, ocular pressure, CB1

## Abstract

Nearly half a century has passed since the demonstration that cannabis and its chief psychoactive component Δ^9^-THC lowers intraocular pressure (IOP). Elevated IOP remains the chief hallmark and therapeutic target for glaucoma, a condition that places millions at risk of blindness. It is likely that Δ^9^-THC exerts much of its IOP-lowering effects via the activation of CB1 cannabinoid receptors. However, the initial promise of CB1 as a target for treating glaucoma has not thus far translated into a credible therapeutic strategy. We have recently shown that blocking monoacylglycerol lipase (MAGL), an enzyme that breaks the endocannabinoid 2-arachidonoyl glycerol (2-AG), substantially lowers IOP. Another strategy is to develop cannabinoid CB1 receptor agonists that are optimized for topical application to the eye. Recently we have reported on a controlled-deactivation approach where the “soft” drug concept of enzymatic deactivation was combined with a “depot effect” that is commonly observed with Δ^9^-THC and other lipophilic cannabinoids. This approach allowed us to develop novel cannabinoids with a predictable duration of action and is particularly attractive for the design of CB1 activators for ophthalmic use with limited or no psychoactive effects. We have tested a novel class of compounds using a combination of electrophysiology in autaptic hippocampal neurons, a well-characterized model of endogenous cannabinoid signaling, and measurements of IOP in a mouse model. We now report that AM7410 is a reasonably potent and efficacious agonist at CB1 in neurons and that it substantially (30%) lowers IOP for as long as 5 h after a single topical treatment. This effect is absent in CB1 knockout mice. Our results indicate that the direct targeting of CB1 receptors with controlled-deactivation ligands is a viable approach to lower IOP in a murine model and merits further study in other model systems.

## 1. Introduction

**Figure 1:**
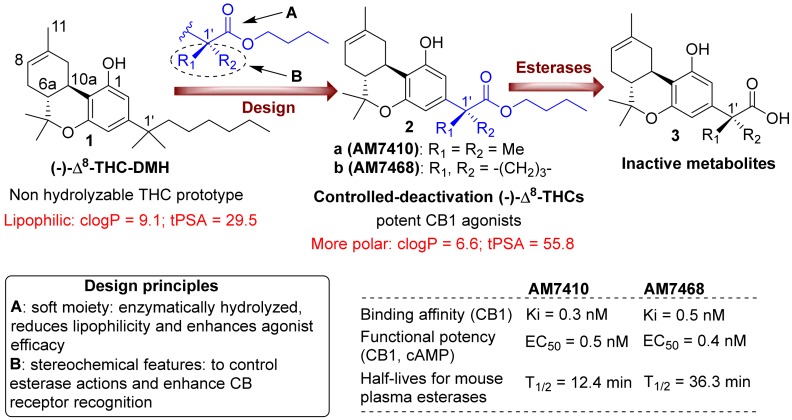
Top panel: design of first-generation side chain carboxylated cannabinoid analogs, with controllable deactivation and increasing polarity, and structures of the prototype (-)-Δ^8^-THC-DMH and inactive metabolites. Lower panel: design principles and biological activity data for representative analogs.

Nearly half a century has passed since the demonstration that cannabis and its chief psychoactive component Δ^9^-THC lowers intraocular pressure (IOP) [1]. Elevated IOP remains the chief hallmark and therapeutic target for glaucoma, a condition that places millions at risk of blindness. It is likely that Δ^9^-THC exerts much of its IOP-lowering effects via the activation of the cannabinoid signaling system (reviewed in [2]), particularly CB1 cannabinoid receptors since it is an agonist at CB1 [3] and CB1 activation lowers ocular pressure [4]. However, the initial promise of CB1 as a target for treating glaucoma has not thus far translated into a credible therapeutic strategy. We have recently shown that blocking monoacylglycerol lipase (MAGL), an enzyme that breaks the endocannabinoid 2-arachidonoyl glycerol (2-AG), substantially lowers IOP for 8 h [5]. Another strategy is to develop cannabinoid CB1 receptor agonists that are optimized for topical application to the eye. THC and related synthetic cannabinoids are reported to only modestly and briefly reduce IOP and risk ocular irritation and toxicity, as well as having the potential for unwanted CNS side effects [6,7,8]. Furthermore, the design of drugs for topical ocular application requires improvements in the bioactivity of the compound with a balance of physicochemical properties for enhanced corneal permeability and ocular bioavailability [7,9]. In this regard, the major problem with the currently known classical cannabinoids is their high lipophilicity (e.g., cLogP > 9 for (-)-Δ^8^-THC-DMH, **1**, Figure 1), and this needs to be improved while maintaining or enhancing in vivo efficacy. Recently, we reported on a controlled-deactivation approach where the “soft” drug concept of enzymatic deactivation was combined with a “depot effect” which is related to the compound’s lipophilicity as well as its tissue distribution and retention [10,11,12,13]. Specifically, we have shown that the incorporation of a metabolically labile carboxy ester group (soft spot) at strategic positions within the THC structure leads to potent and efficacious CB1 agonists (**2**, Figure 1) that are bioconverted to inactive metabolites (**3**) by plasma esterases. Importantly, the rate of hydrolytic cleavage can be accordingly modulated using stereochemical features adjacent to the ester group (enzymatic effect) [10,11]. The depot effect is dependent on the compound’s polar characteristics and can be regulated by varying its log *P* and polar surface area (PSA) values [10,11,13]. This controlled-deactivation approach allowed us to develop novel cannabinoids with a predictable duration of action and an improved druggability, and it is particularly attractive for the design of CB1 activators for ophthalmic use with limited or no psychoactive effects. These potent CB1 agonists are expected to exhibit greater ocular tissue exposure because of their enhanced polarity, and after achieving their therapeutic effect in the eye, they will be deactivated in the blood circulation by esterases.

We have tested this novel class of compounds using a combination of electrophysiology in autaptic hippocampal neurons, a well-characterized model of endogenous cannabinoid signaling, and measurements of IOP in a normotensive mouse model. 

## 2. Methods

### 2.1. Animals

Experiments were conducted at the Indiana University campus. All mice used for IOP experiments were handled according to the guidelines of the Indiana University animal care committee (ethics committee name: BIACUC, protocol number: 16-007). Mice (age 3–8 months) were kept on a 12 h (06:00–18:00) light–dark cycle and fed *ad libitum*. Only male mice were used for these experiments and were obtained from Envigo (Indianapolis, IN, USA) or were kindly provided by Dr. Ken Mackie (Indiana University, Bloomington, IN, USA). The mice were C57BL/6J (C57) strain except CB1^−/−^ mice that were on a CD1 strain background. We have previously shown that mice on a CD1 background see a drop in ocular pressure upon topical treatment with CB1 cannabinoid agonists WIN55212 and CP55940 that are absent in CB1 knockouts [4]. Mice were allowed to acclimatize to the animal care facility for at least a week prior to their use in experiments. CB1^−/−^ mice were kindly provided by Dr. Ken Mackie. The knockouts are global knockouts. CB1^−/−^ animals were originally received from Dr. Catherine Ledent (Catholic University, Leuven, Belgium) as heterozygotes [14]. 

### 2.2. Intraocular Pressure Measurements

The IOP was measured in mice by rebound tonometry using a Tonolab (Icare Finland Oy, Helsinki, Finland). This instrument uses a light plastic-tipped probe to briefly make contact with the cornea; after the probe encounters the eye the instrument measures the speed at which the probe rebounds in order to calculate IOP.

To obtain reproducible IOP measurements, mice were anesthetized with isoflurane (3% induction). The anesthetized mouse was then placed on a platform in a prone position, where anesthesia was maintained with 2% isoflurane. Baseline IOP measurements were taken in both eyes. A ‘measurement’ consisted of the average value of six readings. One eye was then treated with the drug dissolved in Tocrisolve (Tocris Biosciences, Bristol, UK), a soya-based solvent [15], 5 μL final volume applied topically) while the other eye was treated with the vehicle. The animal was then allowed to recover. After an hour, the animal was again anesthetized as above. The IOP was then measured in the drug-treated and the vehicle-treated contralateral eye. 

The IOP measurements following drug administration were analyzed by paired *t*-tests comparing drug-treated eyes to the contralateral vehicle-treated eyes. In animals that were injected with the drug, the ocular pressures of animals were compared with those of vehicle-injected animals and compared using an unpaired *t*-test.

### 2.3. Hippocampal Culture Preparation

Mouse hippocampal neurons isolated from the CA1–CA3 region were cultured on microislands as described previously [16,17]. Neurons were obtained from animals (age postnatal day 0–2) and plated onto a feeder layer of hippocampal astrocytes that had been laid down previously [18]. Cultures were grown in high-glucose (20 mM) Dulbecco’s Modified Eagle’s Medium (DMEM) that contained 10% horse serum, had no mitotic inhibitors, and was used for recordings after eight days in culture and for no more than three hours after removal from the culture medium.

### 2.4. Electrophysiology

When a single neuron is grown on a small island of permissive substrate, it forms synapses—or “autapses”—onto itself. All experiments were performed on isolated autaptic neurons. Whole cell voltage-clamp recordings from autaptic neurons were carried out at room temperature using an Axopatch 200A amplifier (Molecular Devices, Sunnyvale, CA, USA). The extracellular solution contained (in mM) 119 NaCl, 5 KCl, 2.5 CaCl_2_, 1.5 MgCl_2_, 30 glucose, and 20 HEPES. A continuous flow of solution through the bath chamber (~2 mL/min) ensured rapid drug application and clearance. Drugs were typically prepared as stocks, and then they were diluted into an extracellular solution at their final concentration and used on the same day.

Recording pipettes of 1.8–3 MΩ were filled with (in mM) 121.5 KGluconate, 17.5 KCl, 9 NaCl, 1 MgCl_2_, 10 HEPES, 0.2 EGTA, 2 MgATP, and 0.5 LiGTP. The access resistance and the holding current were monitored and only cells with both a stable access resistance and a holding current were included for data analysis. The conventional stimulus protocol was as follows: the membrane potential was held at −70 mV, and the excitatory postsynaptic currents (EPSCs) were evoked every 20 s by triggering an unclamped action current with a 1.0 ms depolarizing step. The resultant evoked waveform consisted of a brief stimulus artifact and a large downward spike representing inward sodium currents, followed by the slower EPSC. The size of the recorded EPSCs was calculated by integrating the evoked current to yield a charge value (in pC). Calculating the charge value in this manner yields an indirect measure of the amount of neurotransmitter released while minimizing the effects of cable distortion on currents generated far from the site of the recording electrode (the soma). Data were acquired at a sampling rate of 5 kHz.

The depolarisation suppression of excitation (DSE) stimuli was as follows: After establishing a 10–20 s 0.5 Hz baseline, the DSE was evoked by depolarizing to 0 mV for 50 ms, 100 ms, 300 ms, 500 ms, 1 s, 3 s, and 10 s, followed in each case by the resumption of a 0.5 Hz stimulus protocol for 20–80+ s, allowing EPSCs to recover to baseline values. This approach allowed us to determine the sensitivity of the synapses to DSE induction. To allow for a comparison, baseline values (prior to the DSE stimulus) were normalized to one. DSE inhibition values were presented as fractions of 1, i.e., a 50% inhibition from the baseline response was 0.50 ± standard error of the mean. The *x*-axis of DSE depolarization–response curves are log-scale seconds of the duration of the depolarization used to elicit the DSE. Depolarization–response curves were obtained to determine the pharmacological properties of endogenous 2-AG signaling by depolarizing neurons for progressively longer durations (50 ms, 100 ms, 300 ms, 500 ms, 1 s, 3 s, and 10 s). The data were fitted with a nonlinear regression, allowing for a calculation of an ED50, the effective dose or duration of depolarization at which a 50% inhibition is achieved. Statistical significance in these curves was taken as non-overlapping 95% confidence intervals.

## 3. Results

### 3.1. AM7410 as a Controlled-Deactivation CB1 Ligand

Through the controlled-deactivation ligand development project we have identified the 1′-*gem*-dimethyl analog AM7410, which exhibits remarkably high affinities for both CB1 and CB2 receptors (Figure 1). The ligand is susceptible to enzymatic hydrolysis by plasma esterases while its metabolite (AM7408) is inactive at the CB receptors. In further in vitro and in vivo experiments, AM7410 was shown to be a potent CB1 receptor agonist and exhibited CB1-mediated hypothermic and analgesic effects.

### 3.2. AM7410 Is a Potent and Efficacious Ligand at CB1 Receptors

**Figure 2:**
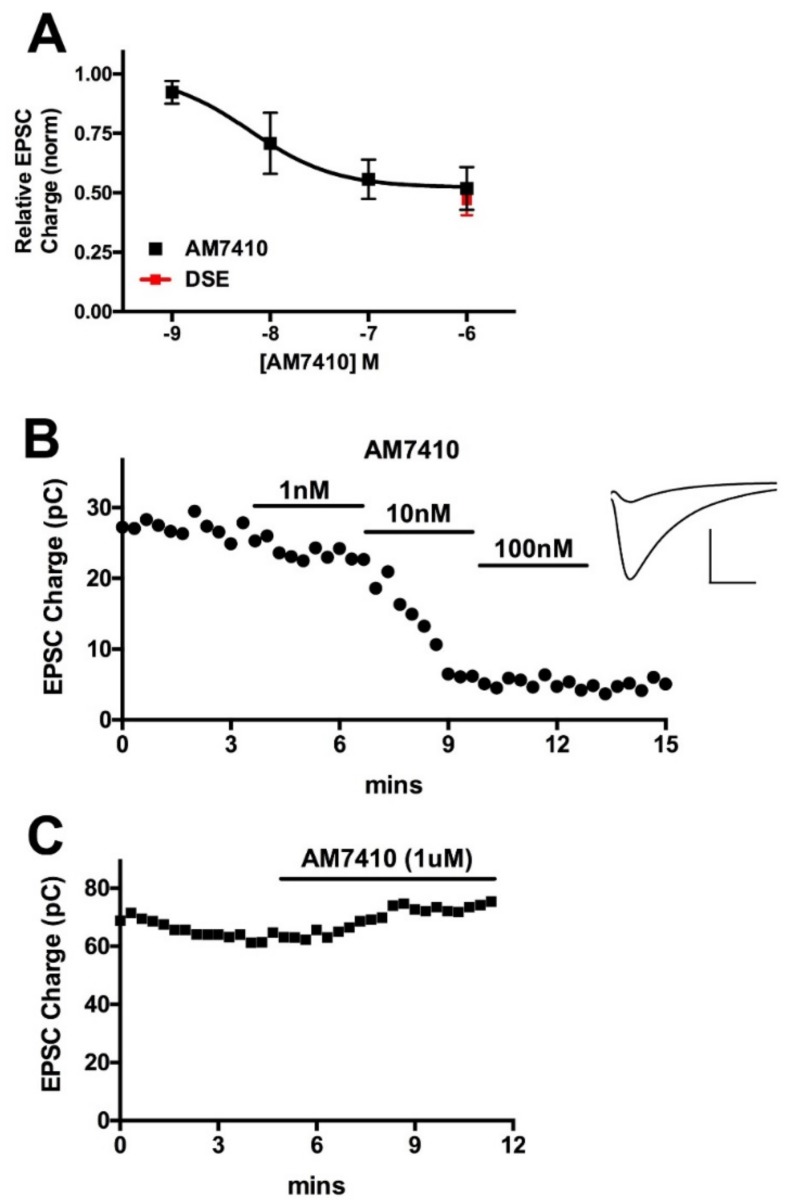
AM7410 is a potent and efficacious CB1 receptor agonist. (**A**) AM7410 inhibits excitatory neurotransmission in autaptic hippocampal neurons in a concentration-dependent manner. Maximal inhibition is similar to maximal inhibition from DSE in the same neurons; (**B**) Time course shows time course of inhibition of EPSCs after treatment with various concentrations of AM7410. Inset shows sample EPSCs before treatment and after treatment with 100 nM AM7410; (**C**) Sample time course shows absence of AM7410 effect in CB1^−/−^ neuron.

We tested the activity of AM7410 in an endogenous neuronal model of cannabinoid signaling. Autaptic hippocampal neurons are a simple, well-characterized preparation that comprises a full circuit of cannabinoid signaling since these neurons produce 2-AG post-synaptically [19], which then acts on presynaptic CB1 receptors, which in turn suppress neurotransmitter release [20]. This retrograde synaptic plasticity is induced by a brief depolarization of the neuron (see Methods) and is termed depolarization induced suppression of excitation or inhibition (DSE/DSI) depending on the neurotransmitter in question [21]. The suppression of neurotransmitter release by a study drug can thus be compared to the suppression elicited by endogenously released 2-AG. As shown in Figure 2, we tested AM7410 at 1 nM, 10 nM, 100 nM, and 1 uM (*n* = 6 at each concentration), which yielded an EC50 of 6.2 nM, which is comparable to our results for WIN55212 [20]. The maximal inhibition due to treatment with AM7410 was similar to that for the maximal inhibition of neurotransmission seen with DSE (relative EPSC charge after AM7410: 0.52 ± 0.07, *n* = 6; after DSE: 0.48 ± 0.07, *n* = 6). Thus, AM7410 is both a potent and efficacious activator of CB1 cannabinoid receptors in this neuronal system. 

### 3.3. AM7410 Lowers Ocular Pressure in a Normotensive Mouse Model

We tested whether the topical application of AM7410 would lower intraocular pressure using rebound tonometry in a normotensive mouse model. The mouse is an established model system for the study of IOP [22,23], offering access to assorted genetic mutants such as CB1 knockout mice, among other things. We have made use of this system in several studies of the regulation of ocular pressure by endocannabinoids [5,24]. 

**Figure 3:**
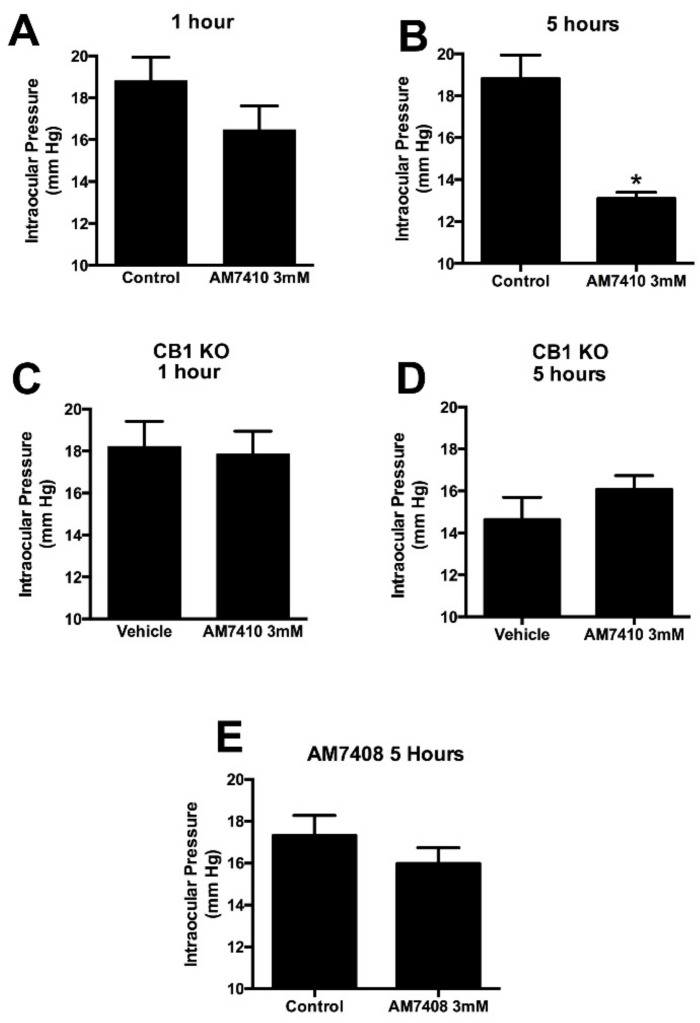
AM7410 lowers intraocular pressure in a normotensive model. (**A**,**B**) AM7410 applied topically at 3 mM lowers ocular pressure at 5 h post-treatment; (**C**,**D**) The effect of AM7410 on ocular pressure is absent in CB1 knockout (KO) mice; (**E**) The inactive metabolite AM7408 does not lower ocular pressure at 5 h. *, *p* < 0.05 by paired *t*-test.

We found that AM7410 applied topically at 3 mM in mice did not lower IOP at 1 h but reduced IOP by 30% at 5 h relative to vehicle-treated contralateral eyes (Figure 3A,B, *n* = 7, 7). This effect was absent in CB1 knockout mice (Figure 3C,D, *n* = 8, 8). We also tested the inactive metabolite of AM7410, AM7408, at 5 h and found it to have no effect (Figure 3E, *n* = 8). 3 mM was chosen because the cornea represents a formidable barrier to drug penetration, necessitating high topical concentrations to assure that a sufficient amount of a given drug penetrates to the intraocular target. Therapeutic topical concentrations of approved glaucoma drugs range as high as 2% (e.g., dorzolamide [25]).

## 4. Discussion

Our chief findings are that the controlled-deactivation CB1 ligand AM7410 is a potent and efficacious agonist at CB1 in a neuronal model of endogenous cannabinoid signaling and that this compound, when applied topically in a normotensive mouse model, substantially reduces intraocular pressure (IOP) by 30% 5 h after treatment, while its metabolite has no effect. 

There are currently six classes of drugs available for the treatment of glaucoma through the lowering of ocular pressure. Not all patients respond to a given drug, and each drug has its own side effect profile (including itching, burning, redness, and altered eye color) that proves to be intolerable in some patients. In addition, because glaucoma is a lifelong disease requiring one or more treatments with eye drops each day, patients develop tolerance to some treatments. There is therefore a continued need for the development of new treatments to effectively lower ocular pressure, but it is generally held that the bar for entry of a new compound is high, with the expectation that a new entrant substantially lowers IOP. AM7410 represents an example of a cannabinoid-based compound that fulfills this expectation, with a 30% drop in IOP in a normotensive model. 

The eye is an ideal setting for the use of a controlled-deactivation ligand. Designed to be rapidly metabolized in the bloodstream, this compound can be applied topically and is allowed to cross the cornea into the ocular chamber where it acts. Once it enters the capillary beds of various ocular tissues, the compound exerts its pharmacological action and is converted to AM7408, a compound that did not lower IOP when applied topically (Figure 2). It may be possible to regulate the duration of the effect of a controlled-deactivation ligand by a rational molecular design of the ligand, thus “tuning” the duration of action. This is supported, for example, by in vitro data comparing the half-lives for plasma esterases of the 1′-gem-dimethyl analog AM7410 and its bulkier 1′-cyclobutyl counterpart AM7468 (Figure 1), as well as by in vivo work we published earlier [10,11]. An additional advantage of the controlled-deactivation CB1 agonists is that they allow structural modifications to produce very polar cannabinoids that are expected to exhibit higher solubility in eye drop preparations as well as in the tears, which results in an increased ocular absorption and ocular drug bioavailability [9,10,13].

In summary, we find that the controlled-deactivation CB1 ligand AM7410 is a potent and efficacious agonist at CB1 in neurons and that it substantially (30%) lowers IOP at least 5 h after a single topical treatment. Our results indicate that the direct targeting of CB1 receptors with controlled-deactivation ligands is a viable approach to lower IOP in a murine model and merits further study in other model systems.
